# Perspectives on Current Challenges and Opportunities for Bovine Viral Diarrhoea Virus Eradication in Australia and New Zealand

**DOI:** 10.3390/pathogens7010014

**Published:** 2018-01-22

**Authors:** Michael P. Reichel, Sasha R. Lanyon, Fraser I. Hill

**Affiliations:** 1 College of Veterinary Medicine and Life Sciences, City University of Hong Kong, Tat Chee Avenue, Kowloon Tong, Hong Kong, China; fraser.hill@cityu.edu.hk; 2 School of Animal and Veterinary Sciences, Roseworthy Campus, University of Adelaide, Roseworthy, SA 5371, Australia; sasha.lanyon@adelaide.edu.au

**Keywords:** bovine viral diarrhoea virus, Australia, New Zealand, eradication, economics, voluntary, compulsory

## Abstract

This review outlines the history of bovine viral diarrhoea virus (BVDV) and the current situation in Australia and New Zealand. BVDV has been reported as present in cattle from both countries for close to 60 years. It rates as the second most economically significant disease afflicting cattle, and is highly prevalent and spread throughout the beef and dairy industries. While other cattle diseases have been the subject of government control and eradication, infection with BVDV is presently not. Eradication has been undertaken in many other countries and been judged to be a good investment, resulting in positive economic returns. Presently, Australia and New Zealand have adopted a non-compulsory approach to control schemes, initiated and managed by farmers and veterinarians without the ultimate goal of eradication. Moving towards eradication is possible with the infrastructure both countries possess, but will require additional resources, coordination, and funding from stakeholders to move to full eradication.

## 1. Introduction

Bovine viral diarrhoea virus (BVDV) is one of the most economically important pathogens of cattle industries worldwide. Cost estimates of the virus on the performance of cattle from around the world vary from USD 33 to 98 per cow [1], and Australia and New Zealand are not exempt from the cost of disease [2]. In Australia, BVDV has recently been identified as the second most economically significant disease of cattle after tick infection, (with an estimated impact of AUD 114 million *per annum* [3]) and the most important pathogen in southern Australian tick-free zones. In New Zealand, while bovine tuberculosis control is still the most economically significant disease of cattle, it is followed by BVDV as the disease of second importance [4].

Thus, in both countries, BVDV rates as the second most impactful cattle disease, yet is not subject to any concerted, coordinated national mitigation approach. The governments do not control the disease, as they do in many other countries.

Therefore, it seems appropriate and timely to review BVDV and its control options in the context of the Australian and New Zealand situation.

## 2. BVDV—The Basics

BVDV is a *Pestivirus* of the family Flaviviridae, with two recognised types (Type 1 and Type 2), numerous subtypes, and two biotypes (cytopathic and noncytopathic) [5,6]. Disease can be divided into three types of infection; acute, fetal, and persistent, each with characteristic clinical signs and outcomes. Acute (also known as transient) infection occurs in naïve, susceptible animals when they are exposed to the virus for the first time [7]. Fetal infections are the result of transmission of the virus across the placenta, due to acute infection in the pregnant dam. Persistent infections are established when fetal infection occurs prior to fetal development of immunocompetence [8]. Persistently infected (PI) cattle are the source of the majority of new acute and fetal infections, as they are continuously highly infectious, shedding the virus in a wide range of body fluids [9].

### Unique Phylogeny

Unlike many other cattle producing countries, BVDV-2 has never been reported in Australian or New Zealand cattle. Australia is unique in that it appears to host predominantly (>97% of isolates; *n* = 351) a single type 1c BVDV strain [10], while New Zealand isolates are primarily of a 1a BVDV strain [11]. This has been attributed to the geographical isolation of Australia and New Zealand in relation to other cattle producing countries. Compared to the Australian situation, there is greater pestivirus diversity observed in New Zealand [12].

## 3. A New Zealand and Australian Perspective

### 3.1. Cattle Industries

A total of about 25 million cattle are farmed in Australia, consisting of 2.66 million dairy cows and 22.3 million beef cattle on about 47,000 properties [13].

Beef production in Australia uses about 200 million hectares of land. There is a distinct North/South split, with Northern cattle production comprising half of the beef cattle herd, and the Southern cattle production being more intensive (smaller herds/less land use). About 60% of the production is exported as beef or live cattle (largely out of the Northern parts). Cattle and calf production is estimated to be worth approximately AUD 12.7 billion [14]. The Australian national dairy industry consists of 1.5 million milking cows on about 5800 farms, and is estimated to be worth, annually, AUD 4.0 billion (being the third largest agricultural industry after beef and wheat). Two thirds of the dairy farms are located in the southern state of Victoria. About 37% of Australian dairy products are exported, estimated to be worth AUD 3.0 billion to the national economy [15].

In New Zealand, about ten million cattle are farmed, consisting of 6.5 million dairy cows on 11,500 farms, and a total beef population of 3.5 million spread throughout the country [16]. Dairy cattle in New Zealand are seasonally calving, and about 95% graze on pasture year-round.

The beef farming industry and dairy farming industry are closely integrated. Surplus dairy bull calves are reared by beef producers, dairy heifers and dairy cows are grazed on beef farms when not lactating, and sire bulls are shared by both producer groups. New Zealand exports about 95% of its dairy products, earning NZD 12.4 billion in 2016 [17]. Beef products were worth NZD 2.7 billion to the New Zealand economy in 2017 [18].

### 3.2. History of BVDV in Australia and New Zealand

The history and prevalence of BVDV in Australia and New Zealand until 1990 is described in detail in a review by Littlejohns and Horner [19].

### 3.3. Australia

Mucosal disease was first reported in Australia in 1957 [20]. The next documented case of BVDV related clinical symptoms was two years later, in 1959, when severe diarrhoea was observed in a group of South Australian yearling cattle [21]. Specific antibodies against BVDV were first reported in Australian cattle in 1964 [22]. That survey showed that between 13% (in South Australia) and 65% (in Victoria) of individual cattle were seropositive [22]. Another survey at around the same time also demonstrated a high proportion of individual cattle as being seropositive—ranging from 35% in Tasmania to 92% in the Northern Territory. At the herd level, that survey showed that over 60% of herds contained seropositive individuals [23]. Taylor [24] demonstrated that between 82% (in Queensland) and 100% (in South Australia and Western Australia) of herds tested showed evidence of exposure to BVDV. On the other hand, Morton, et al. [25] reported around two-thirds of commercial heifer cohorts with over 50% susceptible individuals prior to mating.

The prevalence of PI cattle in Australia is likely to be similar to the 1.4% estimated by Houe and Meyling [26] for Denmark, as the prevalence of PI individuals amongst calves bled for tick research in the 1990s varied between 0 and 3%, and averaged 0.9% [27].

### 3.4. New Zealand

BVDV was first identified in New Zealand in a serological survey, testing for antibody to BVDV [28]. The first case of mucosal disease was described a year later [29].

Analysis of laboratory submissions, in 1975, found 34% of cattle sera tested positive for neutralising antibodies to BVDV [30]. In 1990, a BVDV antibody seroprevalence of 60% [19] was reported, based on analysis of sera submitted for routine diagnostic testing. Another survey in the mid-1990s also found a seroprevalence of 60% in beef herds [31,32]. BVDV accounted for 3.5% of fetal loss in beef herds in a 2013 survey [33]. While peer-reviewed articles on the prevalence of BVDV are few, company and producer group material highlight the significance of infection [34,35].

### 3.5. Economic Costs of BVDV Infection

BVDV is widely acknowledged to have significant financial impacts in infected herds. Losses stem from ill-thrifty PI animals, reproductive disease, decreased production, poor growth, and increased incidence of other diseases. Epidemic outbreaks of BVDV in naïve herds can be explosive, and typically result from the introduction of the virus (usually a PI animal) into a highly susceptible population. The losses are self-limiting, as an increase in herd immunity will limit consequences in following years. However, even in endemically infected herds, in which a high level of immunity is common, consistent low-level losses result in substantial (often unrecognised) losses for many years. Recent estimates provide the cost of BVDV to the Australian cattle industry at approximately AU$114 million each year (Meat and Livestock, Australia) [3].

Losses in NZ dairy herds have been estimated at ranging from NZD 35 [2] to NZD 87 [36] per cow per year in a then average sized (*n* = 393) infected dairy herd. Estimates of the annual losses for NZ dairy farmers are around NZD 127 million, with predicted annual losses of an average of NZD 70,000 for each average-sized infected herd [35].

### 3.6. Vaccination Options

No modified live vaccines are available in either country. In Australia, only one vaccine containing inactivated Bega and Trangie strains of BVDVa is available. In New Zealand, a single vaccine containing an inactivated antigen of cytopathic C-86 strain of BVDVb is used.

### 3.7. Bovine Serum Albumin

Australia is restricted to importing bovine serum albumin (BSA) from New Zealand, Canada and the United States, to avoid the risks of foot and mouth disease [37]. New Zealand only imports BSA from Australia [38] and is an exporter of BSA to other countries. These restrictions were implemented to prevent importation of bovine spongiform encephalopathy (BSE) prions [39], but do not address the risks of the importation of BVDV, and remains an area for both countries to address.

## 4. Opportunities and Challenges for Control

### 4.1. Cross Species Infections/Reservoirs

Historically, *Pestiviruses* were named after the species they were originally isolated from; bovine pestivirus (BVDV) from cattle, ovine pestivirus (border disease virus (BDV)) from sheep, and classical swine fever virus (CSFV) from pigs. Recently however, *Pestiviruses*, particularly BVDV and BDV, have been reported to infect a large number of livestock and wildlife species [40,41].

Worldwide, infections with BVDV have been reported in cattle, sheep, goats, alpaca, camels, deer, rabbits, and a wide range of wildlife species. Similarly, infections with BDV have been reported in sheep, cattle, goats, deer and pigs. Antibodies to BVDV and BVDV antigen have been identified in the sera of sheep [42,43,44], alpacas [45,46,47], goats [48,49], deer [50,51], eland [52], mousedeer [53,54], and pigs [55].

However, reservoir hosts, both wild and domesticated, have not proved troublesome for control programs in Europe [56]. As such, Australia’s feral and native animal populations are unlikely to present a substantial threat to systematic BVDV control. However, if nationwide eradication were the goal, the possible impact of both domestic and feral species (including sheep, goats, buffalo, pigs, deer [19], camels, and alpacas [57]), which have been shown to be susceptible to BVDV infection, may need to be considered in both countries. Water buffalo (*Bubalus bubalis*) in northern Australia only show a low prevalence of BVDV infection, in fact, the level of antibodies present suggest that these few reactors might be non-specific [58]. Alpacas seem readily infected, i.e., seroconvert, yet show minimal clinical signs and BVDV infection in them might go unnoticed (Evans et al., in press, 2018).

Reports of a bull found to be persistently infected with border disease virus [59] and isolation of border disease virus from an Australian bovid [10] demonstrate that sheep-to-cattle pestivirus transmission is possible. Littlejohns and Horner [19] discussed the seroprevalence of BVDV neutralising antibodies in Australian sheep populations, and concluded that interspecies transmission was not likely to be common under field conditions.

Recent studies of BVDV-1c infection in sheep in Australia found that the development of BVDV PI lambs is rare, and that their survival is poor [60,61]. These young PI lambs did not infect other BVDV naïve cattle or sheep (Evans et al. 2017, in preparation). Recent serological surveys in South Australia of close to 900 breeding ewes on 29 properties only revealed antibodies to border disease virus (BDV), but none to BVDV (Evans et al., in press, 2018). It is likely that sheep and other non-bovine species pose very little threat to the persistence of BVDV in cattle populations in these two countries.

### 4.2. Awareness/Misconceptions

If mitigation options are to be considered for BVDV, then farmer compliance would be a primary concern [62]. The need for farmer education is clear. In fact, Lindberg and Alenius [63] affirm that education alone is sufficient to achieve BVDV eradication. As BVDV has no zoonotic potential, social pressure on farmers is minimal, and not sufficient to motivate them to be involved in control schemes [64]. Financial arguments, however can be mounted for well-supported control programs, with both the recent Swiss and Norwegian BVDV mitigation programmes reported to be economically beneficial [65,66,67]. Noticeable progress in the first year of a control program is necessary to maintain dedication amongst farmers and veterinarians, and prevent disease control fatigue [68]. Many of the most successful BVDV control programs (such as the Swedish program [69]) have been initiated by the farming community. By contrast, in New Zealand, the veterinary community has taken the lead in efforts to control BVDV (www.controlbvd.org.nz). In Australia, some movement towards BVDV control was originally made by a BVDV Technical Advisory Group (https://www.bvdvaustralia.com.au/), however, activity has decreased, and the website is outdated. More recently, new management guidelines for the management of BVDV in both the dairy and beef cattle industries have been issued by the Australian Cattle Veterinarians, a special interest group of the Australian Veterinary Association (BVDV Bovine Viral Diarrhoea Virus Management Guide—Beef and Dairy Edition, Australian Cattle Veterinarians (2015)). Preliminary results from a survey of farmer attitudes to BVDV and its control in South Australia suggest that while interest is high, awareness is low, with around 30% of respondents indicating that they do not know any facts about BVDV (Lanyon et al. unpublished data, 2013). These results suggest that an initial education program would be valuable.

### 4.3. Animal Welfare Aspects of BVDV Infection

BVDV infection has a significant effect on the economics of farming, but there is also the effect on the welfare and morale of the farmer as they deal with affected animals. Another consideration is the effect of BVDV infection on the welfare of affected cattle.

From fetus to adult, many infected cattle will die, others will appear apparently normal, though many fail to thrive, while continually shedding infectious viral particles, contributing to survival of the virus, and reinfection of other cattle.

Mucosal disease only develops in persistently infected cattle, and is inevitably fatal after a period of severe clinical disease. Necrosis of keratinocytes in the *stratum spinosum* leads to disruption of intercellular junctions in the keratinised epithelium of the skin, muzzle, oral cavity, oesophagus, rumen, reticulum, and omasum [70]. Normal wear and tear at the epithelial surface leads to erosion and ulceration of the weakened surface, exposing underlying connective tissues. Leakage of fluid from the denuded surface of the gastrointestinal tract results in diarrhoea and dehydration, while bacterial infection and inflammation at the exposed sites results in secondary septicaemia. Death may occur within a few days or be protracted and take a few weeks. Widespread effects on the gastrointestinal tract would induce pain and suffering in affected animals, avoidable by control and eradication of BVDV.

### 4.4. Approaches to Management

BVDV is one disease that veterinarians and farmers can prevent and eliminate. Accurate diagnostic tests, able to be applied to individuals or pools of animal samples have provided the means to detect persistently infected animals. Scandinavian countries showed eradication is possible, based on antibody testing, resulting in improvement of the health of their cattle populations. Switzerland showed that the virus can quickly be removed from the dairy industry by an all-out, concerted effort in a very short period of time [71]. Effects on animal health can be avoided if the cycle of virus spread is broken by finding and removing persistently infected cattle from the population. Austria, Britain, Denmark, Finland, France, Germany, Ireland, Italy, the Netherlands, Norway, Scotland, Slovenia, Sweden, and Switzerland have country or region-wide eradication programs developing or underway.

As a disease with a unique means of spread, where removal of persistently infected cattle leads to elimination of the virus, BVDV is one disease where control or eradication provides an opportunity to improve the health, and therefore the welfare of the cattle population.

### 4.5. Mitigation Options

Some European countries have opted for nationwide eradication with government funded and coordinated programmes. Control efforts are generally considered beneficial, as shown by modelling after the successful Styrian (Austria) and Swiss Dairy BVDV eradication effort [67,72]. While the Austrian evaluation suggested an overall (albeit small) economic benefit, the compulsory phase of eradication was making a loss. However, the authors found with their modelling that there are benefits subsequent to eradication, such as improved producer prices and increased exports.

Both New Zealand and Australia have opted, thus far, for voluntary control on individual farms with education of veterinarians and farmers a key foundation.

There are three phases to eradication; an initial phase of identifying herds likely to be infected, a clearance phase where individual PI cattle are identified and removed, and an ongoing surveillance phase combined with biosecurity, aimed at preventing re-infection [63].

For eradication to be successful, compulsory control may be mandatory, and require legislation to induce testing by reluctant farmers. Suitable infrastructure needs to be in place before embarking on disease control programmes. Australia and New Zealand are in a strong position to support a BVDV control program with these pre-existing infrastructure resources.

Both countries have systems in place for the permanent identification of livestock. In New Zealand, there is a national animal identification and tracing (NAIT) system, introduced under the NAIT Act 2012 [73]. Livestock movement in Australia is monitored by the National Livestock Identification Scheme. All cattle are individually identifiable by an electronic ear tag or rumen bolus [74], and BVDV infection status information could be linked to this [75].

An extensive animal health network exists in both New Zealand and Australia. In both countries, there are government veterinary diagnostic laboratories supported by an extensive network of private diagnostic laboratories with the capacity to test the large number of samples requiring collection. Testing may need to be paid for by the farmers, or subsidized by industry bodies or government to encourage all farmers to participate.

Another factor in favour of successful eradication of BVDV in both countries is their isolation and geography, as Australia and New Zealand’s international biosecurity is facilitated by their geographical location and respective island status.

Nationwide disease eradication from cattle populations has been achieved and maintained in both countries in the past, under schemes such as the Brucellosis and Tuberculosis Eradication Campaign in Australia and the Brucellosis eradication campaign in New Zealand. Aided by ongoing biosecurity controls on cattle movements into the respective countries, it should be possible to maintain BVDV freedom, once achieved.

### 4.6. Discussion and Conclusions

As a disease with a unique means of spread through persistently infected individuals in a herd, elimination of the virus is readily achieved by removing those animals. Currently available diagnostic tests can quickly identify those individuals. BVDV is one disease where control or eradication provides an opportunity to improve the health, and therefore the welfare of the cattle population, while decreasing production losses. Australia and New Zealand have the infrastructure, knowledge, skills, and logistics to undertake eradication, but need the concerted will of farmers guided by veterinarians, veterinary authorities, and government, to move from voluntary control to full eradication.

Sources and manufacturers

- Pestigard^®^ Zoetis Australia Pty Ltd., Level 6, 5 Rider Blvd, Rhodes, NSW 2138, Australia
- Bovilis^®^ MSD Animal Health, 33 Whakatiki St, Upper Hutt, New Zealand.
